# Supplementary material for: Resolving the full spectrum of human genome variation using Linked-Reads
Source: Genome Res. 2019 Apr;29(4):635–45. doi: 10.1101/gr.234443.118 (PMC6442396; doi:10.1101/gr.234443.118)
Supplement: Supplemental Material [file supp_gr.234443.118_Supplemental_longranger-2.2.zip › longranger-2.2.tar.bz2!/lr2.2-public/lib/stan/cmdstan/stan/doc/api/html/classstan_1_1mcmc_1_1chains.html]

Stan: stan::mcmc::chains< RNG > Class Template Reference


|  |  |
| --- | --- |
|  | Stan  2.5.0  probability, sampling & optimization |


- Main Page
- Related Pages
- Namespaces
- Classes
- Files

- Class List
- Class Index
- Class Hierarchy
- Class Members

All Classes Namespaces Files Functions Variables Typedefs Enumerations Enumerator Friends Macros Pages

- stan
- mcmc
- chains

Public Member Functions |
List of all members

stan::mcmc::chains< RNG > Class Template Reference

An `mcmc::chains` object stores parameter names and dimensionalities along with samples from multiple chains.
More...

`#include <chains.hpp>`

|  |  |
| --- | --- |
| Public Member Functions | |
|  | chains (const Eigen::Matrix< std::string, Eigen::Dynamic, 1 > &param\_names) |
|  | |
|  | chains (const std::vector< std::string > &param\_names) |
|  | |
|  | chains (const stan::io::stan\_csv &stan\_csv) |
|  | |
| int | num\_chains () const |
|  | |
| int | num\_params () const |
|  | |
| const Eigen::Matrix  < std::string, Eigen::Dynamic, 1 > & | param\_names () const |
|  | |
| const std::string & | param\_name (int j) const |
|  | |
| int | index (const std::string &name) const |
|  | |
| void | set\_warmup (const int chain, const int warmup) |
|  | |
| void | set\_warmup (const int warmup) |
|  | |
| const Eigen::VectorXi & | warmup () const |
|  | |
| int | warmup (const int chain) const |
|  | |
| int | num\_samples (const int chain) const |
|  | |
| int | num\_samples () const |
|  | |
| int | num\_kept\_samples (const int chain) const |
|  | |
| int | num\_kept\_samples () const |
|  | |
| void | add (const int chain, const Eigen::MatrixXd &sample) |
|  | |
| void | add (const Eigen::MatrixXd &sample) |
|  | |
| void | add (const std::vector< std::vector< double > > &sample) |
|  | Convert a vector of vector<double> to Eigen::MatrixXd. More... |
|  | |
| void | add (const stan::io::stan\_csv &stan\_csv) |
|  | |
| Eigen::VectorXd | samples (const int chain, const int index) const |
|  | |
| Eigen::VectorXd | samples (const int index) const |
|  | |
| Eigen::VectorXd | samples (const int chain, const std::string &name) const |
|  | |
| Eigen::VectorXd | samples (const std::string &name) const |
|  | |
| double | mean (const int chain, const int index) const |
|  | |
| double | mean (const int index) const |
|  | |
| double | mean (const int chain, const std::string &name) const |
|  | |
| double | mean (const std::string &name) const |
|  | |
| double | sd (const int chain, const int index) const |
|  | |
| double | sd (const int index) const |
|  | |
| double | sd (const int chain, const std::string &name) const |
|  | |
| double | sd (const std::string &name) const |
|  | |
| double | variance (const int chain, const int index) const |
|  | |
| double | variance (const int index) const |
|  | |
| double | variance (const int chain, const std::string &name) const |
|  | |
| double | variance (const std::string &name) const |
|  | |
| double | covariance (const int chain, const int index1, const int index2) const |
|  | |
| double | covariance (const int index1, const int index2) const |
|  | |
| double | covariance (const int chain, const std::string &name1, const std::string &name2) const |
|  | |
| double | covariance (const std::string &name1, const std::string &name2) const |
|  | |
| double | correlation (const int chain, const int index1, const int index2) const |
|  | |
| double | correlation (const int index1, const int index2) const |
|  | |
| double | correlation (const int chain, const std::string &name1, const std::string &name2) const |
|  | |
| double | correlation (const std::string &name1, const std::string &name2) const |
|  | |
| double | quantile (const int chain, const int index, const double prob) const |
|  | |
| double | quantile (const int index, const double prob) const |
|  | |
| double | quantile (int chain, const std::string &name, double prob) const |
|  | |
| double | quantile (const std::string &name, const double prob) const |
|  | |
| Eigen::VectorXd | quantiles (int chain, int index, const Eigen::VectorXd &probs) const |
|  | |
| Eigen::VectorXd | quantiles (int index, const Eigen::VectorXd &probs) const |
|  | |
| Eigen::VectorXd | quantiles (int chain, const std::string &name, const Eigen::VectorXd &probs) const |
|  | |
| Eigen::VectorXd | quantiles (const std::string &name, const Eigen::VectorXd &probs) const |
|  | |
| Eigen::Vector2d | central\_interval (int chain, int index, double prob) const |
|  | |
| Eigen::Vector2d | central\_interval (int index, double prob) const |
|  | |
| Eigen::Vector2d | central\_interval (int chain, const std::string &name, double prob) const |
|  | |
| Eigen::Vector2d | central\_interval (const std::string &name, double prob) const |
|  | |
| Eigen::VectorXd | autocorrelation (const int chain, const int index) const |
|  | |
| Eigen::VectorXd | autocorrelation (int chain, const std::string &name) const |
|  | |
| Eigen::VectorXd | autocovariance (const int chain, const int index) const |
|  | |
| Eigen::VectorXd | autocovariance (int chain, const std::string &name) const |
|  | |
| double | effective\_sample\_size (const int index) const |
|  | |
| double | effective\_sample\_size (const std::string &name) const |
|  | |
| double | split\_potential\_scale\_reduction (const int index) const |
|  | |
| double | split\_potential\_scale\_reduction (const std::string &name) const |
|  | |

## Detailed Description

### template<typename RNG = boost::random::ecuyer1988> class stan::mcmc::chains< RNG >

An `mcmc::chains` object stores parameter names and dimensionalities along with samples from multiple chains.

**Synchronization**: For arbitrary concurrent use, the read and write methods need to be read/write locked. Multiple writers can be used concurrently if they write to different chains. Readers for single chains need only be read/write locked with writers of that chain. For reading across chains, full read/write locking is required. Thus methods will be classified as global or single-chain read or write methods.

**Storage Order**: Storage is column/last-index major.

Definition at line 55 of file chains.hpp.

## Constructor & Destructor Documentation

template<typename RNG = boost::random::ecuyer1988>

|  |  |  |  |  |  |
| --- | --- | --- | --- | --- | --- |
| stan::mcmc::chains< RNG >::chains | ( | const Eigen::Matrix< std::string, Eigen::Dynamic, 1 > & | *param\_names* | ) |  |

inline

Definition at line 326 of file chains.hpp.

template<typename RNG = boost::random::ecuyer1988>

|  |  |  |  |  |  |
| --- | --- | --- | --- | --- | --- |
| stan::mcmc::chains< RNG >::chains | ( | const std::vector< std::string > & | *param\_names* | ) |  |

inline

Definition at line 329 of file chains.hpp.

template<typename RNG = boost::random::ecuyer1988>

|  |  |  |  |  |  |
| --- | --- | --- | --- | --- | --- |
| stan::mcmc::chains< RNG >::chains | ( | const stan::io::stan\_csv & | *stan\_csv* | ) |  |

inline

Definition at line 334 of file chains.hpp.

## Member Function Documentation

template<typename RNG = boost::random::ecuyer1988>

|  |  |  |  |
| --- | --- | --- | --- |
| void stan::mcmc::chains< RNG >::add | ( | const int | *chain*, |
|  |  | const Eigen::MatrixXd & | *sample* |
|  | ) |  |  |

inline

Definition at line 402 of file chains.hpp.

template<typename RNG = boost::random::ecuyer1988>

|  |  |  |  |  |  |
| --- | --- | --- | --- | --- | --- |
| void stan::mcmc::chains< RNG >::add | ( | const Eigen::MatrixXd & | *sample* | ) |  |

inline

Definition at line 437 of file chains.hpp.

template<typename RNG = boost::random::ecuyer1988>

|  |  |  |  |  |  |
| --- | --- | --- | --- | --- | --- |
| void stan::mcmc::chains< RNG >::add | ( | const std::vector< std::vector< double > > & | *sample* | ) |  |

inline

Convert a vector of vector<double> to Eigen::MatrixXd.

This method is added for the benefit of software wrapping Stan (e.g., PyStan) so that it need not additionally wrap Eigen.

Definition at line 453 of file chains.hpp.

template<typename RNG = boost::random::ecuyer1988>

|  |  |  |  |  |  |
| --- | --- | --- | --- | --- | --- |
| void stan::mcmc::chains< RNG >::add | ( | const stan::io::stan\_csv & | *stan\_csv* | ) |  |

inline

Definition at line 466 of file chains.hpp.

template<typename RNG = boost::random::ecuyer1988>

|  |  |  |  |
| --- | --- | --- | --- |
| Eigen::VectorXd stan::mcmc::chains< RNG >::autocorrelation | ( | const int | *chain*, |
|  |  | const int | *index* |
|  | ) |  | const |

inline

Definition at line 656 of file chains.hpp.

template<typename RNG = boost::random::ecuyer1988>

|  |  |  |  |
| --- | --- | --- | --- |
| Eigen::VectorXd stan::mcmc::chains< RNG >::autocorrelation | ( | int | *chain*, |
|  |  | const std::string & | *name* |
|  | ) |  | const |

inline

Definition at line 660 of file chains.hpp.

template<typename RNG = boost::random::ecuyer1988>

|  |  |  |  |
| --- | --- | --- | --- |
| Eigen::VectorXd stan::mcmc::chains< RNG >::autocovariance | ( | const int | *chain*, |
|  |  | const int | *index* |
|  | ) |  | const |

inline

Definition at line 665 of file chains.hpp.

template<typename RNG = boost::random::ecuyer1988>

|  |  |  |  |
| --- | --- | --- | --- |
| Eigen::VectorXd stan::mcmc::chains< RNG >::autocovariance | ( | int | *chain*, |
|  |  | const std::string & | *name* |
|  | ) |  | const |

inline

Definition at line 669 of file chains.hpp.

template<typename RNG = boost::random::ecuyer1988>

|  |  |  |  |
| --- | --- | --- | --- |
| Eigen::Vector2d stan::mcmc::chains< RNG >::central\_interval | ( | int | *chain*, |
|  |  | int | *index*, |
|  |  | double | *prob* |
|  | ) |  | const |

inline

Definition at line 625 of file chains.hpp.

template<typename RNG = boost::random::ecuyer1988>

|  |  |  |  |
| --- | --- | --- | --- |
| Eigen::Vector2d stan::mcmc::chains< RNG >::central\_interval | ( | int | *index*, |
|  |  | double | *prob* |
|  | ) |  | const |

inline

Definition at line 636 of file chains.hpp.

template<typename RNG = boost::random::ecuyer1988>

|  |  |  |  |
| --- | --- | --- | --- |
| Eigen::Vector2d stan::mcmc::chains< RNG >::central\_interval | ( | int | *chain*, |
|  |  | const std::string & | *name*, |
|  |  | double | *prob* |
|  | ) |  | const |

inline

Definition at line 646 of file chains.hpp.

template<typename RNG = boost::random::ecuyer1988>

|  |  |  |  |
| --- | --- | --- | --- |
| Eigen::Vector2d stan::mcmc::chains< RNG >::central\_interval | ( | const std::string & | *name*, |
|  |  | double | *prob* |
|  | ) |  | const |

inline

Definition at line 651 of file chains.hpp.

template<typename RNG = boost::random::ecuyer1988>

|  |  |  |  |
| --- | --- | --- | --- |
| double stan::mcmc::chains< RNG >::correlation | ( | const int | *chain*, |
|  |  | const int | *index1*, |
|  |  | const int | *index2* |
|  | ) |  | const |

inline

Definition at line 570 of file chains.hpp.

template<typename RNG = boost::random::ecuyer1988>

|  |  |  |  |
| --- | --- | --- | --- |
| double stan::mcmc::chains< RNG >::correlation | ( | const int | *index1*, |
|  |  | const int | *index2* |
|  | ) |  | const |

inline

Definition at line 574 of file chains.hpp.

template<typename RNG = boost::random::ecuyer1988>

|  |  |  |  |
| --- | --- | --- | --- |
| double stan::mcmc::chains< RNG >::correlation | ( | const int | *chain*, |
|  |  | const std::string & | *name1*, |
|  |  | const std::string & | *name2* |
|  | ) |  | const |

inline

Definition at line 578 of file chains.hpp.

template<typename RNG = boost::random::ecuyer1988>

|  |  |  |  |
| --- | --- | --- | --- |
| double stan::mcmc::chains< RNG >::correlation | ( | const std::string & | *name1*, |
|  |  | const std::string & | *name2* |
|  | ) |  | const |

inline

Definition at line 584 of file chains.hpp.

template<typename RNG = boost::random::ecuyer1988>

|  |  |  |  |
| --- | --- | --- | --- |
| double stan::mcmc::chains< RNG >::covariance | ( | const int | *chain*, |
|  |  | const int | *index1*, |
|  |  | const int | *index2* |
|  | ) |  | const |

inline

Definition at line 551 of file chains.hpp.

template<typename RNG = boost::random::ecuyer1988>

|  |  |  |  |
| --- | --- | --- | --- |
| double stan::mcmc::chains< RNG >::covariance | ( | const int | *index1*, |
|  |  | const int | *index2* |
|  | ) |  | const |

inline

Definition at line 555 of file chains.hpp.

template<typename RNG = boost::random::ecuyer1988>

|  |  |  |  |
| --- | --- | --- | --- |
| double stan::mcmc::chains< RNG >::covariance | ( | const int | *chain*, |
|  |  | const std::string & | *name1*, |
|  |  | const std::string & | *name2* |
|  | ) |  | const |

inline

Definition at line 559 of file chains.hpp.

template<typename RNG = boost::random::ecuyer1988>

|  |  |  |  |
| --- | --- | --- | --- |
| double stan::mcmc::chains< RNG >::covariance | ( | const std::string & | *name1*, |
|  |  | const std::string & | *name2* |
|  | ) |  | const |

inline

Definition at line 565 of file chains.hpp.

template<typename RNG = boost::random::ecuyer1988>

|  |  |  |  |  |  |
| --- | --- | --- | --- | --- | --- |
| double stan::mcmc::chains< RNG >::effective\_sample\_size | ( | const int | *index* | ) | const |

inline

Definition at line 674 of file chains.hpp.

template<typename RNG = boost::random::ecuyer1988>

|  |  |  |  |  |  |
| --- | --- | --- | --- | --- | --- |
| double stan::mcmc::chains< RNG >::effective\_sample\_size | ( | const std::string & | *name* | ) | const |

inline

Definition at line 683 of file chains.hpp.

template<typename RNG = boost::random::ecuyer1988>

|  |  |  |  |  |  |
| --- | --- | --- | --- | --- | --- |
| int stan::mcmc::chains< RNG >::index | ( | const std::string & | *name* | ) | const |

inline

Definition at line 356 of file chains.hpp.

template<typename RNG = boost::random::ecuyer1988>

|  |  |  |  |
| --- | --- | --- | --- |
| double stan::mcmc::chains< RNG >::mean | ( | const int | *chain*, |
|  |  | const int | *index* |
|  | ) |  | const |

inline

Definition at line 502 of file chains.hpp.

template<typename RNG = boost::random::ecuyer1988>

|  |  |  |  |  |  |
| --- | --- | --- | --- | --- | --- |
| double stan::mcmc::chains< RNG >::mean | ( | const int | *index* | ) | const |

inline

Definition at line 506 of file chains.hpp.

template<typename RNG = boost::random::ecuyer1988>

|  |  |  |  |
| --- | --- | --- | --- |
| double stan::mcmc::chains< RNG >::mean | ( | const int | *chain*, |
|  |  | const std::string & | *name* |
|  | ) |  | const |

inline

Definition at line 510 of file chains.hpp.

template<typename RNG = boost::random::ecuyer1988>

|  |  |  |  |  |  |
| --- | --- | --- | --- | --- | --- |
| double stan::mcmc::chains< RNG >::mean | ( | const std::string & | *name* | ) | const |

inline

Definition at line 514 of file chains.hpp.

template<typename RNG = boost::random::ecuyer1988>

|  |  |  |  |  |
| --- | --- | --- | --- | --- |
| int stan::mcmc::chains< RNG >::num\_chains | ( |  | ) | const |

inline

Definition at line 340 of file chains.hpp.

template<typename RNG = boost::random::ecuyer1988>

|  |  |  |  |  |  |
| --- | --- | --- | --- | --- | --- |
| int stan::mcmc::chains< RNG >::num\_kept\_samples | ( | const int | *chain* | ) | const |

inline

Definition at line 391 of file chains.hpp.

template<typename RNG = boost::random::ecuyer1988>

|  |  |  |  |  |
| --- | --- | --- | --- | --- |
| int stan::mcmc::chains< RNG >::num\_kept\_samples | ( |  | ) | const |

inline

Definition at line 395 of file chains.hpp.

template<typename RNG = boost::random::ecuyer1988>

|  |  |  |  |  |
| --- | --- | --- | --- | --- |
| int stan::mcmc::chains< RNG >::num\_params | ( |  | ) | const |

inline

Definition at line 344 of file chains.hpp.

template<typename RNG = boost::random::ecuyer1988>

|  |  |  |  |  |  |
| --- | --- | --- | --- | --- | --- |
| int stan::mcmc::chains< RNG >::num\_samples | ( | const int | *chain* | ) | const |

inline

Definition at line 380 of file chains.hpp.

template<typename RNG = boost::random::ecuyer1988>

|  |  |  |  |  |
| --- | --- | --- | --- | --- |
| int stan::mcmc::chains< RNG >::num\_samples | ( |  | ) | const |

inline

Definition at line 384 of file chains.hpp.

template<typename RNG = boost::random::ecuyer1988>

|  |  |  |  |  |  |
| --- | --- | --- | --- | --- | --- |
| const std::string& stan::mcmc::chains< RNG >::param\_name | ( | int | *j* | ) | const |

inline

Definition at line 352 of file chains.hpp.

template<typename RNG = boost::random::ecuyer1988>

|  |  |  |  |  |
| --- | --- | --- | --- | --- |
| const Eigen::Matrix<std::string, Eigen::Dynamic, 1>& stan::mcmc::chains< RNG >::param\_names | ( |  | ) | const |

inline

Definition at line 348 of file chains.hpp.

template<typename RNG = boost::random::ecuyer1988>

|  |  |  |  |
| --- | --- | --- | --- |
| double stan::mcmc::chains< RNG >::quantile | ( | const int | *chain*, |
|  |  | const int | *index*, |
|  |  | const double | *prob* |
|  | ) |  | const |

inline

Definition at line 589 of file chains.hpp.

template<typename RNG = boost::random::ecuyer1988>

|  |  |  |  |
| --- | --- | --- | --- |
| double stan::mcmc::chains< RNG >::quantile | ( | const int | *index*, |
|  |  | const double | *prob* |
|  | ) |  | const |

inline

Definition at line 593 of file chains.hpp.

template<typename RNG = boost::random::ecuyer1988>

|  |  |  |  |
| --- | --- | --- | --- |
| double stan::mcmc::chains< RNG >::quantile | ( | int | *chain*, |
|  |  | const std::string & | *name*, |
|  |  | double | *prob* |
|  | ) |  | const |

inline

Definition at line 597 of file chains.hpp.

template<typename RNG = boost::random::ecuyer1988>

|  |  |  |  |
| --- | --- | --- | --- |
| double stan::mcmc::chains< RNG >::quantile | ( | const std::string & | *name*, |
|  |  | const double | *prob* |
|  | ) |  | const |

inline

Definition at line 601 of file chains.hpp.

template<typename RNG = boost::random::ecuyer1988>

|  |  |  |  |
| --- | --- | --- | --- |
| Eigen::VectorXd stan::mcmc::chains< RNG >::quantiles | ( | int | *chain*, |
|  |  | int | *index*, |
|  |  | const Eigen::VectorXd & | *probs* |
|  | ) |  | const |

inline

Definition at line 606 of file chains.hpp.

template<typename RNG = boost::random::ecuyer1988>

|  |  |  |  |
| --- | --- | --- | --- |
| Eigen::VectorXd stan::mcmc::chains< RNG >::quantiles | ( | int | *index*, |
|  |  | const Eigen::VectorXd & | *probs* |
|  | ) |  | const |

inline

Definition at line 610 of file chains.hpp.

template<typename RNG = boost::random::ecuyer1988>

|  |  |  |  |
| --- | --- | --- | --- |
| Eigen::VectorXd stan::mcmc::chains< RNG >::quantiles | ( | int | *chain*, |
|  |  | const std::string & | *name*, |
|  |  | const Eigen::VectorXd & | *probs* |
|  | ) |  | const |

inline

Definition at line 615 of file chains.hpp.

template<typename RNG = boost::random::ecuyer1988>

|  |  |  |  |
| --- | --- | --- | --- |
| Eigen::VectorXd stan::mcmc::chains< RNG >::quantiles | ( | const std::string & | *name*, |
|  |  | const Eigen::VectorXd & | *probs* |
|  | ) |  | const |

inline

Definition at line 621 of file chains.hpp.

template<typename RNG = boost::random::ecuyer1988>

|  |  |  |  |
| --- | --- | --- | --- |
| Eigen::VectorXd stan::mcmc::chains< RNG >::samples | ( | const int | *chain*, |
|  |  | const int | *index* |
|  | ) |  | const |

inline

Definition at line 479 of file chains.hpp.

template<typename RNG = boost::random::ecuyer1988>

|  |  |  |  |  |  |
| --- | --- | --- | --- | --- | --- |
| Eigen::VectorXd stan::mcmc::chains< RNG >::samples | ( | const int | *index* | ) | const |

inline

Definition at line 483 of file chains.hpp.

template<typename RNG = boost::random::ecuyer1988>

|  |  |  |  |
| --- | --- | --- | --- |
| Eigen::VectorXd stan::mcmc::chains< RNG >::samples | ( | const int | *chain*, |
|  |  | const std::string & | *name* |
|  | ) |  | const |

inline

Definition at line 494 of file chains.hpp.

template<typename RNG = boost::random::ecuyer1988>

|  |  |  |  |  |  |
| --- | --- | --- | --- | --- | --- |
| Eigen::VectorXd stan::mcmc::chains< RNG >::samples | ( | const std::string & | *name* | ) | const |

inline

Definition at line 498 of file chains.hpp.

template<typename RNG = boost::random::ecuyer1988>

|  |  |  |  |
| --- | --- | --- | --- |
| double stan::mcmc::chains< RNG >::sd | ( | const int | *chain*, |
|  |  | const int | *index* |
|  | ) |  | const |

inline

Definition at line 518 of file chains.hpp.

template<typename RNG = boost::random::ecuyer1988>

|  |  |  |  |  |  |
| --- | --- | --- | --- | --- | --- |
| double stan::mcmc::chains< RNG >::sd | ( | const int | *index* | ) | const |

inline

Definition at line 522 of file chains.hpp.

template<typename RNG = boost::random::ecuyer1988>

|  |  |  |  |
| --- | --- | --- | --- |
| double stan::mcmc::chains< RNG >::sd | ( | const int | *chain*, |
|  |  | const std::string & | *name* |
|  | ) |  | const |

inline

Definition at line 526 of file chains.hpp.

template<typename RNG = boost::random::ecuyer1988>

|  |  |  |  |  |  |
| --- | --- | --- | --- | --- | --- |
| double stan::mcmc::chains< RNG >::sd | ( | const std::string & | *name* | ) | const |

inline

Definition at line 530 of file chains.hpp.

template<typename RNG = boost::random::ecuyer1988>

|  |  |  |  |
| --- | --- | --- | --- |
| void stan::mcmc::chains< RNG >::set\_warmup | ( | const int | *chain*, |
|  |  | const int | *warmup* |
|  | ) |  |  |

inline

Definition at line 364 of file chains.hpp.

template<typename RNG = boost::random::ecuyer1988>

|  |  |  |  |  |  |
| --- | --- | --- | --- | --- | --- |
| void stan::mcmc::chains< RNG >::set\_warmup | ( | const int | *warmup* | ) |  |

inline

Definition at line 368 of file chains.hpp.

template<typename RNG = boost::random::ecuyer1988>

|  |  |  |  |  |  |
| --- | --- | --- | --- | --- | --- |
| double stan::mcmc::chains< RNG >::split\_potential\_scale\_reduction | ( | const int | *index* | ) | const |

inline

Definition at line 687 of file chains.hpp.

template<typename RNG = boost::random::ecuyer1988>

|  |  |  |  |  |  |
| --- | --- | --- | --- | --- | --- |
| double stan::mcmc::chains< RNG >::split\_potential\_scale\_reduction | ( | const std::string & | *name* | ) | const |

inline

Definition at line 697 of file chains.hpp.

template<typename RNG = boost::random::ecuyer1988>

|  |  |  |  |
| --- | --- | --- | --- |
| double stan::mcmc::chains< RNG >::variance | ( | const int | *chain*, |
|  |  | const int | *index* |
|  | ) |  | const |

inline

Definition at line 534 of file chains.hpp.

template<typename RNG = boost::random::ecuyer1988>

|  |  |  |  |  |  |
| --- | --- | --- | --- | --- | --- |
| double stan::mcmc::chains< RNG >::variance | ( | const int | *index* | ) | const |

inline

Definition at line 538 of file chains.hpp.

template<typename RNG = boost::random::ecuyer1988>

|  |  |  |  |
| --- | --- | --- | --- |
| double stan::mcmc::chains< RNG >::variance | ( | const int | *chain*, |
|  |  | const std::string & | *name* |
|  | ) |  | const |

inline

Definition at line 542 of file chains.hpp.

template<typename RNG = boost::random::ecuyer1988>

|  |  |  |  |  |  |
| --- | --- | --- | --- | --- | --- |
| double stan::mcmc::chains< RNG >::variance | ( | const std::string & | *name* | ) | const |

inline

Definition at line 546 of file chains.hpp.

template<typename RNG = boost::random::ecuyer1988>

|  |  |  |  |  |
| --- | --- | --- | --- | --- |
| const Eigen::VectorXi& stan::mcmc::chains< RNG >::warmup | ( |  | ) | const |

inline

Definition at line 372 of file chains.hpp.

template<typename RNG = boost::random::ecuyer1988>

|  |  |  |  |  |  |
| --- | --- | --- | --- | --- | --- |
| int stan::mcmc::chains< RNG >::warmup | ( | const int | *chain* | ) | const |

inline

Definition at line 376 of file chains.hpp.

---

The documentation for this class was generated from the following file:

- src/stan/mcmc/chains.hpp


---

[ Stan Home Page ]

*© 2011–2014,
Stan Development Team.*
